# On the Emergence of Autonomous Chemical Systems through Dissipation Kinetics

**DOI:** 10.3390/life13112171

**Published:** 2023-11-06

**Authors:** Addy Pross, Robert Pascal

**Affiliations:** 1 Department of Chemistry, Ben-Gurion University of the Negev, Be’er-Sheva 8410501, Israel; pross@bgu.ac.il; 2 PIIM, Institut Origines, Aix-Marseille Université—CNRS, Service 232, Saint Jérôme, Ave Escadrille Normandie Niemen, 13013 Marseille, France

**Keywords:** origin of life, evolution, autocatalysis, irreversibility, dynamic kinetic stability, emerging autonomous systems

## Abstract

This work addresses the kinetic requirements for compensating the entropic cost of self-organization and natural selection, thereby revealing a fundamental principle in biology. Metabolic and evolutionary features of life cannot therefore be separated from an origin of life perspective. Growth, self-organization, evolution and dissipation processes need to be metabolically coupled and fueled by low-entropy energy harvested from the environment. The evolutionary process requires a reproduction cycle involving out-of-equilibrium intermediates and kinetic barriers that prevent the reproductive cycle from proceeding in reverse. Model analysis leads to the unexpectedly simple relationship that the system should be fed energy with a potential exceeding a value related to the ratio of the generation time to the transition state lifetime, thereby enabling a process mimicking natural selection to take place. Reproducing life’s main features, in particular its Darwinian behavior, therefore requires satisfying constraints that relate to time and energy. Irreversible reaction cycles made only of unstable entities reproduce some of these essential features, thereby offering a physical/chemical basis for the possible emergence of autonomy. Such Emerging Autonomous Systems (EASs) are found to be capable of maintaining and reproducing their kind through the transmission of a stable kinetic state, thereby offering a physical/chemical basis for what could be deemed an epigenetic process.

## 1. Introduction

Any reductionist approach toward the life phenomenon could be based on a retrosynthetic dissection into its several sub-systems—its metabolism, its genetic information storage system, and the one enabling compartmentalization. Previously, such an approach has usually focused on the main molecular components of these systems, namely proteins, nucleic acids and phospholipids. Despite considerable progress made in prebiotic chemistry and further developments leading to the identification of pathways for the formation of most biomolecular cell components (building blocks and corresponding oligomers) [1,2,3], this approach does not seem to have provided a solution towards reproducing artificially a chemical system able to express life characteristics. Considering life as an emergent process taking place through the combination of different parts of a system, as normally taken in a reductionist approach [4], leads to difficulties in identifying the pathway by which the main features of life could have emerged. Indeed, the discovery of abiotic chemical processes able to produce amino acids, as reported by Miller [5], was unable to provide a pathway toward the reproduction of life from molecular beginnings. The same conclusion can be drawn from experiments in synthetic biology that demonstrated the possibility of exchanging the genetic material of a cell to generate a different one [6]. This possibility means that a cellular compartment, cleared of genetic carriers but continuing to host an organized arrangement of multiple components, is able to empower an otherwise non-functional artificial genome through metabolic factors that have remained elusive thus far. Thus, the ability to design systems endowed with such a capability seems to be a prerequisite for artificially reproducing the main features of life.

Synthetic prebiotic chemists have traditionally focused their investigations on selected pathways that produce significant yields of biochemical building blocks from pure chemical precursors. However, most experiments carried out in a manner simulating prebiotic environments [7,8,9], together with analyses of abiotic organic matter found in solar system bodies [10], have led to the conclusion that abiotic organic chemistry usually produces a huge diversity of structures, greatly exceeding those present in extant life forms. This observation made in numerous instances constitutes one of the paradoxes [11,12] inherent in the chemical approaches to the origin of life that begin with abiotic chemistry. Moreover, the reasons why biochemically relevant components could have been selected and concentrated is made even more puzzling by considering the fact that no report of successful attempts to build a living organism de novo starting with purified components have been published to date.

Putting aside the traditional reductionist approach based on dissecting life into its molecular components, an alternative approach could be found by considering the kinetic features of living organisms. In this approach, both the molecular components of life, as well as the chemical reaction network responsible for the living state, may have been selected together from the wide diversity of abiotic processes. A kinetic perspective was clearly involved in experiments on the replication of RNA strands that inspired the early analyses of Eigen and co-workers. They revealed the importance of the inherent instability of these replicators, being undermined by an error threshold, and suggested a role for hypercycles in the preservation of genetic information [13,14]. The concept of Dynamic Kinetic Stability (DKS) was also understood as a kinetic phenomenon, one associated with the power of replication as a driving force for the self-organization of life [15,16]. This concept was deduced from the tautological time definition of stability, whereby systems can only evolve spontaneously into a state of increased stability until a point where no further change is possible [17]. Noteworthy, this persistence principle [18,19,20] also operates independently for isolated systems evolving towards the equilibrium state (thermodynamically stable by definition and corresponding to an entropy maximum). Thus, both the drive toward thermodynamic stability and toward DKS can account for the organization of matter in systems that are either close to equilibrium, or contrariwise, maintained far from equilibrium through a continuous supply of free energy [18].

The ability to undergo variability and selection together constitute the two independent properties of replicators needed to fully mimic natural selection, with the former taking place through a non-limited (or at least wide) range of heritable variations. The present investigation analyses the selection capability by considering a model involving out-of-equilibrium systems, able to couple dissipation and reproduction within an autocatalytic cycle. We seek to identify under which conditions a selection process similar to that of natural selection can take place, therefore establishing an evolutionary process towards a state of increased dynamic stability [17]. As living beings, such systems can only express this behavior under far-from-equilibrium conditions maintained by a supply of low-entropy free energy carriers. Accordingly, life requires a non-equilibrium environment in which the dissipation of the energy brought about by low entropy energy carriers can compensate for the development of organized structures, thereby avoiding a violation of the Second Law [21]. Dissipation, namely the conversion of low-entropy energy forms into heat or other degraded forms of energy, is fundamentally irreversible, a fact consistent with the observation that the development of living organisms, even in the cell cycle, one of its more essential processes, takes place in an irreversible manner. This irreversibility requirement is essential, with the consequence that populations of the simplest biological entities (cells, for instance) cannot be considered in the same way as populations of molecular particles, atoms or molecules [22], whose motion in physics is considered as obeying the principle of microscopic reversibility. In this report, the fundamental specificity of biology that rests upon microscopically irreversible processes and conditions under which simple models of autocatalysis can reproduce such behavior are addressed, thereby taking an important step toward simulating the transition towards the living state. Though working on quite simple models of autocatalysis is certainly not sufficient to synthesize life de novo from molecules, given the necessary cooperative processes and other stabilizing interactions that are needed [23,24,25], the present approach may lead to insights into how features of life and selection can be implemented in a system that is as simple as possible, as a prerequisite for dealing with more complex dynamic assemblies.

## 2. Coupling the Dissipation of Energy Carrier Potential with Self-Organization

**Figure 1:**
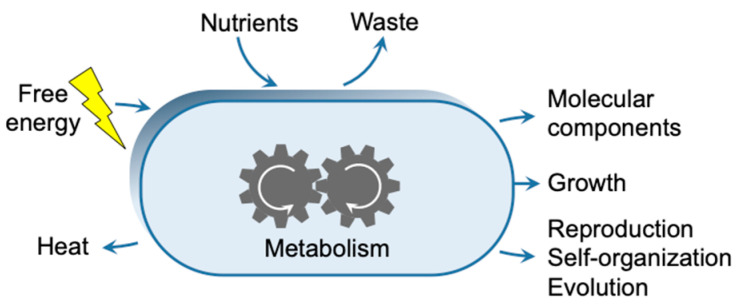
In addition to the synthesis of the cell’s molecular components, its metabolism ensures a functional coupling between, on the one hand, the dissipation of the potential of energy sources and, on the other hand, the development, the reproduction and the evolution of living entities, all of which require them occurring in an irreversible manner in order that evolution remains a historical process directed towards increasing DKS. In this work, lightning bolts are used to picture an input of free energy into the system. In biology, energy carriers are built though catabolism, photosynthesis or the utilization of redox potentials. In abiotic chemistry, the activation process can be of a physical nature (resulting, for instance, from spark discharges or of the absorption of photons), which brings the system to a high-energy state that is quenched at ambient temperature impeding the system to relax to the equilibrium state. Alternatively, chemical activation can take place by reaction with high-energy species, which are held in a far-from-equilibrium state by kinetic barriers [28,29,30,31]. These activated chemicals can be considered to store the potential reached through a prior physical or geophysical activation process within an energy well surrounded by kinetic barriers. The nitrile triple bond can indeed correspond to this definition and its specific reaction with thiols provides an example of how chemical energy can be harvested in a synthetic way [32].

There is no obvious connection between the evolutionary description of biology and the thermodynamic requirements for the self-organization of living organisms. The proposal by Schrödinger [21] that living organisms continually draw “negative entropy” from their environment was highly provocative since it is obviously in contradiction with Boltzmann’s definition of entropy that is incompatible with negative values. However, feeding a system with low-entropy energy and coupling dissipation with its self-organization [2], constitutes an alternative means that avoids any violation of physical laws. This statement expresses the view that the self-organization features associated with life cannot be settled without introducing a functional coupling of growth and evolution with energy dissipation. This coupling should be considered as one of, if not the main role of the metabolism, from an evolutionary perspective (Figure 1). Therefore, the synthesis of molecular components of the cell, which would actually not necessarily be incompatible with a close to equilibrium situation, does not constitute the only function of metabolism, consistently with earlier thoughtful remarks on the fact that the focus of origin of life research should not only consider anabolism [26]. Its additional evolutionary role has independently ensured the maintenance of a non-equilibrium state from the time that life on Earth emerged some 3.5 billion years ago, and, therefore, would have been present as a component of a “minimal metabolism” [27]. The energized character of living entities associated with the irreversibility of dissipation is then intimately responsible for their maintenance and evolutionary features, a connection that needs to be directly addressed. 

Understanding how metabolism is able to couple dissipation with reproduction and evolution using a simple model therefore constitutes an essential step toward an understanding of life’s emergence. This role must be addressed in the same way as other constraints that are associated with its origin and its relationship with the origin of life [33]. An essential consequence of the irreversible character of the development of living entities lies in the fact that it allows a historical evolutionary process to take place. In this process, further changes depend on the preceding states of the system as well as on contingent circumstances. They should proceed in the direction of increased DKS [16] in an out-of-equilibrium environment that must be conserved without interruption to avoid any disruption in the historical process. Most of these changes may fit in with the notion of bifurcation that was introduced to account for the analysis of self-organization in far-from-equilibrium systems [34].

Dissipation corresponds to the conversion of chemical free energy potential into heat or other degraded forms of energy. When dissipation is complete, the relaxed system can no longer evolve spontaneously having reached thermal equilibrium. In the process of feeding living systems with energy, focusing on dissipation would suggest that the amount of heat produced is the determining factor. It has indeed been proposed that the increase in dissipation rates can drive self-organization [35,36,37]. However, this directing role of dissipation rates has been subject to criticism [38,39,40]. Moreover, it is important to consider the difference between heat, the result of dissipation, an extensive quantity and which is additive for subsystems [41], and the energy potential, an intensive quantity [41], the magnitude of which is independent of system size, and which governs the transformations which can take place. The important parameter involved in this process therefore lies in the energy potential that leads to dissipation in a single microscopic event rather than in the amount of heat that could be released through the sum total of several contiguous events.

**Figure 2:**
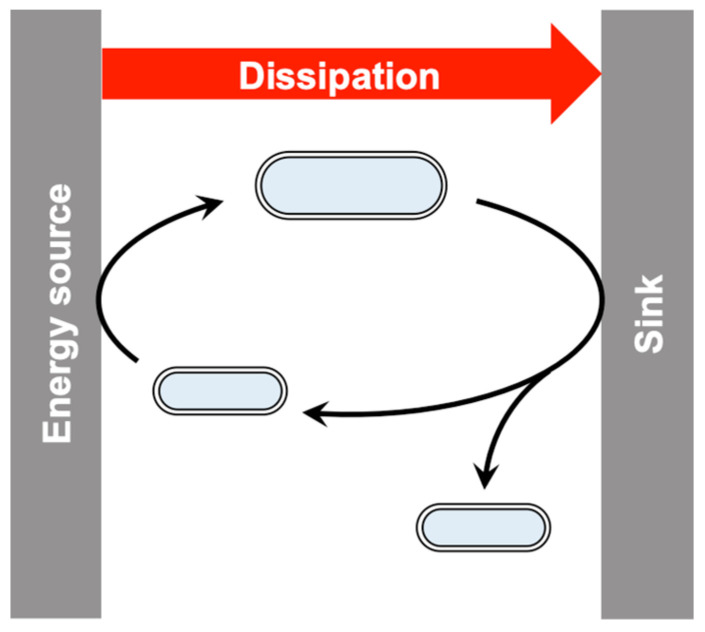
Development and reproduction of a living cell fed from a free energy source (e.g., nutrients in a disequilibrium state present in the environment). The cell cycle contributes to dissipate the energy potential into degraded forms corresponding to an energy sink (e.g., heat or inactivated waste). Note that recycling a cell into its initial state only results in dissipation and that chemical work is realized through the cell cycle just because one extra copy of the living being is produced per round of the cycle. By intimately coupling dissipation with the entropy loss associated with the reproduction of a thermodynamically unstable entity, the cell cycle avoids any violation of the Second Law.

Dissipation takes place when a gradient of energy is present in the environment between a source of high potential (corresponding to low-entropy energy) and a sink corresponding to a degraded form of energy (high-entropy) from which work can no longer be obtained (Figure 2). However, without kinetic barriers the potential of the source would become spontaneously exhausted, rapidly leading to the equilibrium state, without the possibility of alternative pathways. Therefore, such barriers need to be present in order that disequilibrium can be held for significant periods of time [28,29,30,31]. The process through which living cells can multiply without violation of the Second Law in this context is depicted in Figure 2. Indeed, reproduction and dissipation proceed at the same time, being part of a single process, the cell cycle, which must necessarily be consistent with physical principles. This process induces both energy dissipation as well as the realization of chemical work through the formation of an extra cell which itself corresponds to a non-equilibrium entity. It is important, at this stage, to emphasize that living entities must correspond to energized systems and that their existence within an equilibrium environment would lead to their fast decay to the equilibrium state and death. This non-equilibrium requirement can also be understood by considering that any evolutionary process needs single entities to be eliminated (biologically speaking, to die) in order that they can be replaced by improved ones [24]. Therefore, three different elements, namely the metabolic features of life requiring the maintenance of an energized non-equilibrium situation, an evolutionary capability, and a dissipative process, turn out to be intimately linked to each other in constituting a living entity. All depend on the continual presence of disequilibrium of the system with respect to the environment. 

At this stage, two main conclusions can be drawn. The first, that no single molecular entity can bear all of these features, so a process involving different components, and therefore a systems chemistry approach, is required [27,42,43,44,45]. The second, that chemical processes devised to reproduce features of life should therefore simultaneously possess the three essential capabilities specified above. In the following section, these capabilities will be introduced consecutively, indicating how these features could be chemically implemented.

### 2.1. Enabling Thermodynamically Unstable Transient States to Become Kinetically Stable

At equilibrium, thermal agitation populates a given microstate with a probability *p*_i_ that is proportional to an exponential function of the ratio of the energy of this microstate *ε*_i_ to its thermal energy *k*_B_*T* (Equation (1)), as expected for a Boltzmann distribution.

(1) $$p_{i}\;\propto \;e^{\left(-\frac{\varepsilon_{i}}{k_{B}T}\right)}$$

Increasing specifically the concentration of given microstates away from the statistical distribution within the phase space induces a decrease in entropy, since the resulting system is associated with a probability that is lower than that expected for thermal equilibrium. The Second Law disallows such a spontaneous change towards lower probability in isolated systems. Fueling the system through a supply of energy can overcome this limitation while leading subsequently to the dissipation of the corresponding potential energy that is converted into heat together with reagent deactivation. Self-organization processes involving unstable transient intermediates can therefore be obtained in these open systems without any violation of the Second Law since dissipation can compensate for the associated decrease in entropy.

**Figure 3:**
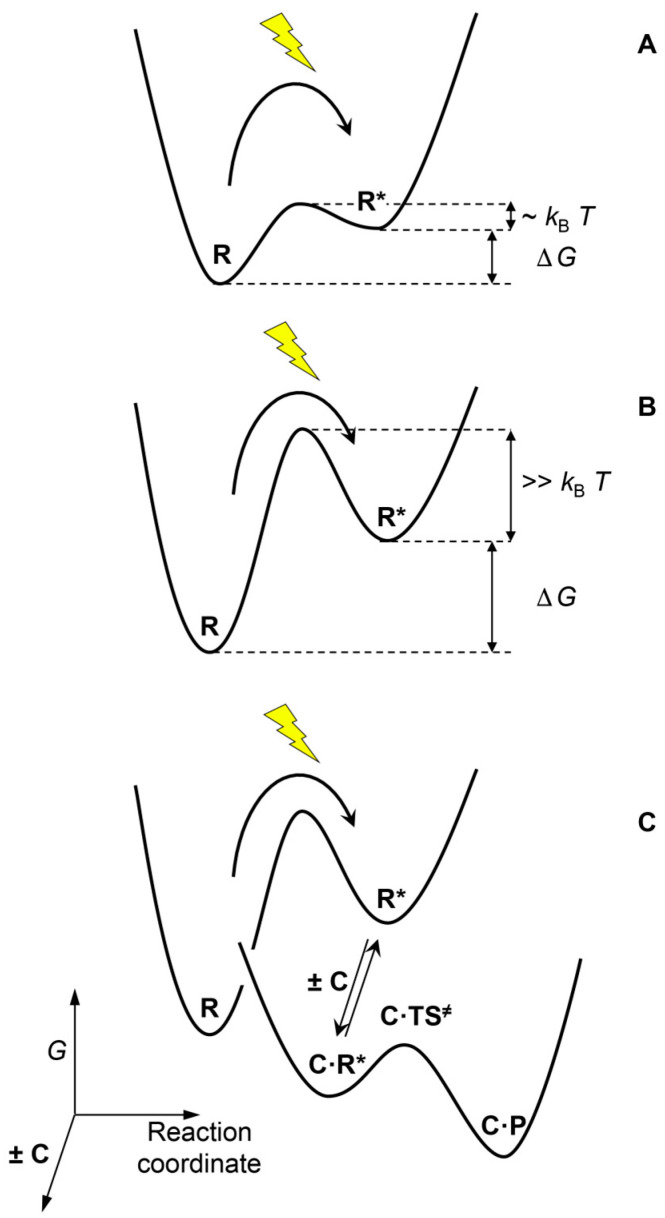
Activating a chemical system can lead to different situations depending on the free energy difference ΔG between the activated state R* (in this work, the asterisk indicates that the molecular entity is not thermodynamically stable and therefore absent at equilibrium) and the inactivated reactant **R** and on the kinetic barrier separating the two states. (**A**) It may lead to an unstable state with no significant lifetime, or (**B**) to a kinetically stable state depending on the height of the barrier separating the activated state from the equilibrium state. (**C**) The existence of kinetic barriers opens the possibility of alternative pathways depending on the presence of additional reactants, as for instance a catalyst **C** forming a complex C·R* with the activated state R* thereby stabilizing the transition state **TS^≠^** leading to product.

The fate of a chemical system that undergoes a perturbation resulting from the supply of energy will depend on its acquired potential, one enabling the system to establish a transient unstable state, and on the presence of kinetic barriers that prevent the system in that unstable state from spontaneously reverting back to the equilibrium state (Figure 3). Perturbations of limited magnitude would only affect rotational or vibrational degrees of freedom in the system (Figure 3A). In condensed phases, energy relaxation accompanying the pathway back to equilibrium from these perturbed states would be rapid since it would only depend on the occurrence of mechanical interaction between molecules. Different behavior would be observed when the activated state is separated from the equilibrium state by kinetic barriers and when thermal agitation is not sufficient to allow rapid crossing of these barriers (Figure 3B). In such cases, a non-equilibrium state could become both populated and persistent, provided the activation process is maintained, consistent with the concept of non-replicative DKS states [46]. Indeed, significant kinetic barriers are often observed when covalent bonds maintain the system within a free energy well. As a result, these bonds usually need to be broken, at least to some degree, before their components are able to react with other partners. Therefore, a molecular architecture bound through covalent bonds can become both kinetically stable as well as thermodynamically activated [47,48]. In this situation the fate of the system will depend on the kinetics associated with reaction pathways connecting them to products rather than on the thermodynamic stability of the final state. Alternative pathways involving other reagents present in the system can become faster than direct reversal into inactivated reactants, and kinetically compete with the spontaneous release of free energy to the environment (Figure 3C). In this way, the potential associated with the perturbation may be used through a process that couples dissipation with the metabolic utilization of free energy (Figure 1).

### 2.2. Dissipative Self-Assembly

**Figure 4:**
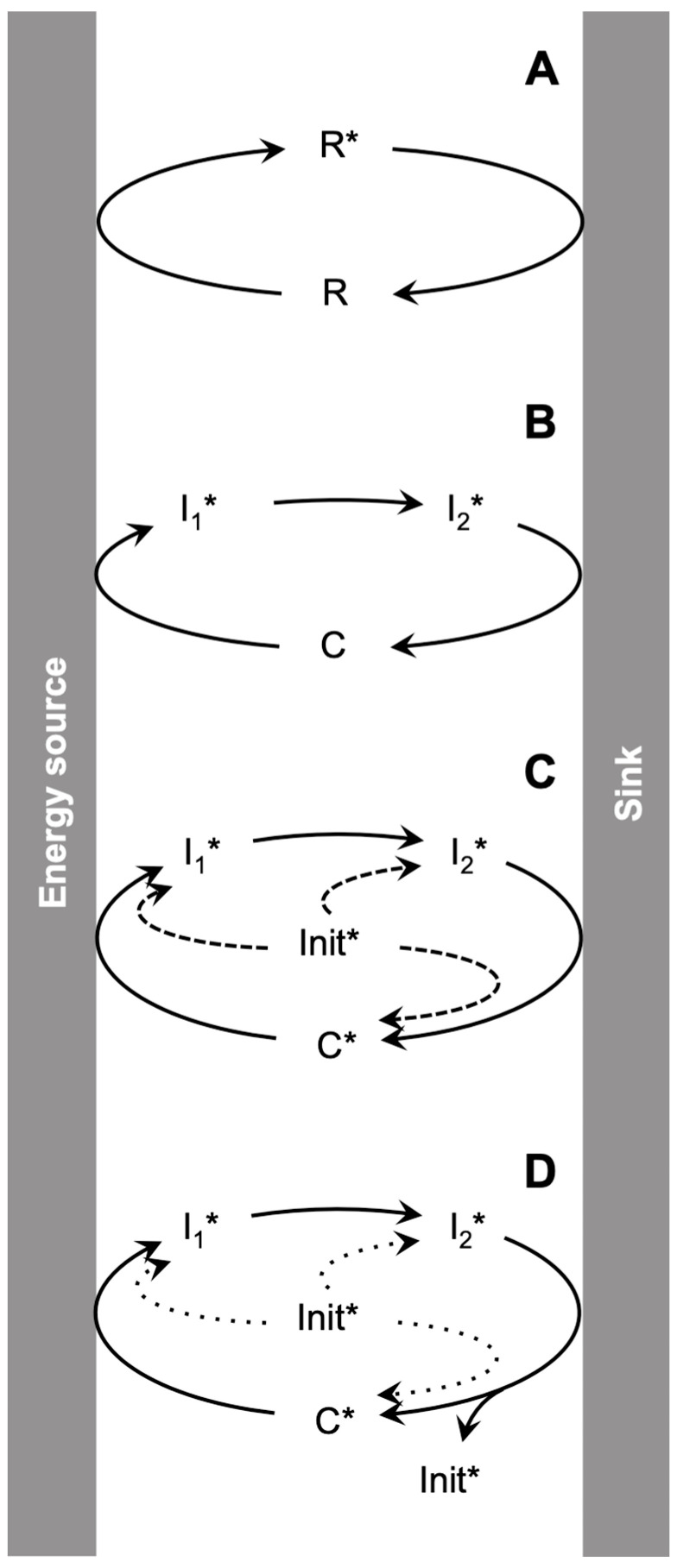
Dissipation associated with chemical processes of increasing complexity occurring in a disequilibrium context involving an energy source and a sink (for simplicity, the addition of reactants and the release of inactivated products have been omitted from this scheme and can be considered as belonging, respectively to the source or the sink). (**A**) The activation of a reagent **R** can lead to the thermodynamically unstable form R* in a kinetically stable state that reverts to the reactant by releasing its energy content as heat. Apart from its energy content, chemical work can be produced and stored in the system only if the activated form R* is specifically subject to an additional process such as polymerization (dissipative self-assembly). (**B**) Process involving a chemically stable catalyst **C**, which, in principle, can be totally regenerated through a catalytic cycle. The activated intermediates I_1_* and I_2_* are formed in a DKS state with the cleavage of these species releasing heat, inactivated products and regenerating the stable catalyst **C**. The whole system cannot be considered as being in a DKS state since **C** corresponds to a ground state and energy is stored in the system only to maintain the DKS concentration of intermediates I_1_* and I_2_*. (**C**) Process involving an unstable catalyst C*. In this case, the usual non-quantitative character of chemical reactions would lead to a breakdown of the catalyst leading to limited turnover numbers except if an energized (activated) initiator Init* is continuously provided into the system. Init* can in principle be any one of the components of the catalytic cycle, C*, I_1_* or I_2_* since reconstituting the whole cycle needs only one of its components. Interestingly, differentiating the initiator from the catalyst illustrates the fact that most of the energy stored in the process does not mandatorily come from the energy source but also from the activated initiator Init*. (**D**) A self-maintained catalytic system corresponding to the definition of an Emerging Autonomous System (EAS) in which the initiator Init* is produced in one extra copy through a reaction cycle which is effectively autocatalytic. In this case, *the whole catalytic cycle* (including C*, I_1_* and I_2_*)**,** rather than specific individual intermediates, becomes a rudimentary autonomous system (at least autonomous from the initiator) and is both DKS-stable and able to store energy derived from some source and to grow at its expense in a way similar to the cell cycle of Figure 2. Interestingly, the activated initiator Init* constitutes a true initiator that needs only to be added once, though the entire system would end up collapsing if the non-equilibrium environment was to dissipate. Restarting the system would then require a new initiation using the *activated* initiator Init*.

An example of self-organization processes coupled to dissipation is that of dissipative self-assembly. Such a process enables the formation of transient macromolecular structures in chemically fueled systems (for instance, by the specific formation of aggregates from the activated form R*, Figure 3 and Figure 4A) [49,50,51]. The principles leading to the formation of these dynamic systems mimicking essential structures in cells have been thoroughly analyzed [52,53]. Systems of this kind can present very different behavior depending on the values of the particular rate constants. Specific properties, including the realization of chemical work through the storage of energy in the molecular assembly, are possible when the system involves *kinetic asymmetry*, a concept initially introduced to explain how imposed fluctuations can drive a system away from equilibrium [54], but that appears to be relevant in the self-organization of life [40]. These kinds of processes, based on kinetics, are responsible for many dynamic self-organization features in biology [52,53] but also in the functioning of molecular machines [55].

### 2.3. Activation Cycles

The notion of kinetic asymmetry implies that the activation of the system and its relaxation toward the equilibrium state at least partly take place through different pathways, seemingly defying the principle of microscopic reversibility [56]. This situation corresponds to that of a unidirectional cycle (Figure 4), which can be attained (without violation of any physical principle) in disequilibrium open systems involving a physical (photochemical, for instance) or chemical (through the effect of high-energy reagents) source of energy, provided an activation process that maintains the system in its far-from-equilibrium state continually operates. A consequence of this approach is that activation is associated with reaction cycles in which the forward and reverse flows are not equivalent, leading to kinetic asymmetry and the resulting establishment of an irreversible cyclic process (Figure 4). A kinetic analysis of the corresponding cycles can be made using specific tools [57].

Indeed, establishing a correspondence between the description of cycles in terms of free energy (including that of transition states) and their kinetic properties is not straightforward. It could turn out to be essential to account in a quantitative way for self-organization in these systems. It has been the topic of investigations in the field of catalysis [58]. Methods that are beyond the scope of the present investigation have also been proposed to represent systems prone to self-organization processes, such as molecular motors or self-assembly processes [59]. However, relevant information can be obtained through a much simpler investigation into the means by which activated reaction cycles can also operate when arising through the effect of an energy gradient. Cyclic arrangements of increasing complexity are depicted in Figure 4, starting from a simple activation cycle in which a reactant **R** is brought to an activated state R* by interaction with an energy source and then deactivated through a spontaneous pathway, releasing heat into the environment (Figure 4A). Regarding the environment in which the energy source is held in a disequilibrium state by kinetic barriers [28,29,30,31], this process can be considered as catalytic by increasing the flux transferred into the sink. It may involve a non-limited number of intermediates like I_1_* or I_2_* constituting a catalytic cycle (Figure 4B). In these catalytic cycles, activated intermediates R*, I_1_* or I_2_*, thermodynamically unstable by definition, are maintained in a steady state corresponding to a state of Dynamic Kinetic Stability (DKS), and their concentration is determined by kinetics. If the disequilibrium is suspended by removing the energy source, the system will evolve towards thermodynamically stable reactant **R** or catalyst **C**. The whole cyclic process would be recovered from those species after re-establishing the non-equilibrium environment. 

However, a very different outcome would be expected by considering a system in which the catalyst C* (Figure 4C,D) is not thermodynamically stable and spontaneously and fully decomposes into inactivated reactants unable to induce any catalytic activity. In this case, the system needs the introduction of an initiator Init* that can be any one of the activated intermediates of the cycle (C*, I_1_* or I_2_* in Figure 4C). Despite the necessary kinetic barriers protecting these energized intermediates [28,29,30,31], unavoidable deactivation processes are likely to take place as a result of side-reactions. Then, the catalytic cycle will proceed allowing finite turnover numbers depending on the kinetics of the side-reactions relative to the turnover time of the catalytic cycle (Figure 4C). In this system all the intermediates of the cycle are maintained in a DKS state and will vanish if the energy source is removed or exhausted. However, the system is not autonomous as its existence depends on an external supply providing one of the intermediates of the catalytic cycle (Init*). 

Autonomy may arise; however, if the catalytic cycle is additionally able to produce the initiator Init* in an amount exceeding that which is consumed during the cycle turnover time (Figure 4D). In such cases, the need to continuously provide the activated initiator Init* is no longer necessary since it is produced autocatalytically by the cycle. Thus, not only the intermediates C*, I_1_* or I_2_* (Figure 4D) are in a DKS state, but the entire system becomes DKS-stable, and importantly, *the system becomes chemically autonomous*, given its independence from an initiator. Any disruption in the energy source, however, would lead the system to collapse without any possibility of restarting unless an initiation event induced by a novel introduction of Init* were to take place. This analysis of the peculiarities of cyclic systems fed from energy sources illustrates the specificity of the reproducing systems that are able to reach DKS states while *being only constituted of entities that are not present at equilibrium*. It also allows for a deeper understanding of the specificity of replicative DKS systems [46] applied to unstable entities that reproduce essential features of living systems, being based on the *energized character* of all the intermediate stages of the cell cycle (Figure 2). The special nature of these systems is worth emphasizing by identifying them as Emerging Autonomous Systems (EASs). This concept is reminiscent of that of Seed-Dependent Autonomous Systems (SDAS) that have been proposed in the independent approach of Baum and coworkers aimed at applying ecological principles to complex networks of reactions in an origin of life context [60,61], but without considering any relationship with dissipation, thermodynamic instability and dynamic kinetic stability. By contrast, our approach is aimed at drawing conclusions by analyzing much simpler systems. In a Synthetic Biology perspective, this analysis is significant in that it describes how an autonomous chemical system could arise spontaneously. Clearly, an understanding of the autonomous nature of all living things would depend on an understanding of how an autonomous chemical system of *any* kind could have emerged from within some non-equilibrium system. In addition, the analysis also suggests that the essential features of life were unlikely to have emerged spontaneously from a mixture of stable reactants or intermediates, and that it would have required an activated initiator or a specific initiating process, beyond the general requirement of a system in thermodynamic disequilibrium. Such features would need to be taken into account in undertaking origin of life studies.

## 3. Looking Back from Evolutionary Biology to Chemical Selection

A driving force for biological evolution was proposed through Darwin’s theory of evolution [62] more than 160 years ago, based on randomly introduced heritable variation and natural selection. This theory was inspired by both wildlife observations and Malthus’s ideas [63] regarding the impossibility of an exponentially growing population taking place within a finite environment. The discovery of the structure of nucleic acids and further developments in molecular biology provided a basis for the genetic mechanisms supporting heredity, completing the modern synthesis of evolutionary biology.

### 3.1. Mimicking Natural Selection in Chemistry

Evolutionary approaches have revealed that these central concepts also apply to artificial systems, as demonstrated by pioneering in vitro experiments on the evolution of RNA strands [64]. However, the corresponding description of evolutionary mechanisms provided no explanation for the pathways by which nucleic acid replication as well as the expression of genetic information could have been established in the emergence of life process. A living entity capable of evolving according to these laws must be equipped with a genetic storage system that allows it to overcome the very limited evolutionary possibilities of autocatalytic sets [65,66]. This condition means that random variation (mutation) could emerge and be transmitted to offspring while undergoing a selection process. The replication of the sequence of a co-polymer composed of several monomers, such as those that exist in nucleic acids, constitutes a straightforward means by which genetically encoded variations can be produced in almost infinite diversity. However, template replicators, for which product inhibition is associated with the increased affinity of dimers formed from the longer strands of ligated products compared to segments, strongly limit their ability to serve as a model of natural selection [67,68,69]. Reinstating exponential growth under these conditions requires recourse to more complex systems involving multiple components [14,24,70,71] in a way more consistent with a systems chemistry approach [27,42,43,44,45]. However, though a molecular system must be present to allow for variation, selection can be analyzed by considering its kinetic features only (as shown, for instance, when considering the competition between two replicators that reproduces features of natural selection [72]). The essence of natural selection is of a kinetic character and is independent of the physical nature of the entities and the structure of the carriers of genetic information. Focusing on kinetic aspects while putting aside the genetic features of life constitutes an oversimplification, as understanding the kinetic features of life also requires the identity of molecular entities to be specified. However, the kinetic principles that underpin natural selection are worthy of independent analysis using simple models, which may additionally be helpful in understanding at which stage Darwinian concepts could have been introduced in the continuous search for improved persistence as a driver of evolution.

### 3.2. Stoichiometric Conditions for Autocatalysis

Though the above analysis shows that the emergence of life may require a dissipative autocatalytic cycle composed only of energized intermediates, some more general conclusions on autocatalysis are worth emphasizing. Autocatalysis (as in, for instance, the system shown in Figure 4D) does not mandatorily involve explicit catalysis of a particular chemical step. It can constitute an emergent property resulting from the occurrence of cyclic networks of reactions rather than from the direct catalytic contribution of a product in its own formation [73]. The formose reaction [74], in which no step can individually be considered catalytic provides a typical example in which autocatalysis results from the architecture of the network rather than from a given chemical step. The description of the various ways through which autocatalysis may be observed has been investigated by a number of groups [60,61,75,76,77,78,79,80,81,82]. The fact that autocatalysis may be more easily detected by studying networks of reactions rather than through the investigation of a single transformation can be considered a key reason why so few examples are available. Therefore, just because synthetic chemists typically perform reactions stepwise in order to maximize yields rather than through a systemic approach, alternative possibilities based on unanticipated pathways could well exist.

An additional requirement, specific to autocatalysis, lies in the fact that more than one stoichiometric unit of catalyst must be released per round of the cycle [77]. All the species involved in the cycle, or in the network for more complex systems, must therefore be protected from breakdown into inactive side-products by kinetic barriers. Accordingly, the time scales for these detrimental processes must exceed the generation time—the mean time lapse separating generations—otherwise all components of the cycle would eventually drop away to zero. This remark emphasizes the importance of kinetic barriers as a requirement for self-organization in chemical systems [28,29,30,31], a point that was already noted above. It also mitigates the preceding observation that autocatalysis can result from large networks of reactions since the realization of the stoichiometric condition may become more difficult to achieve as the size of the network increases owing to the increased likelihood of side reactions.

### 3.3. Achieving Irreversibility in Autocatalysis

**Figure 5:**
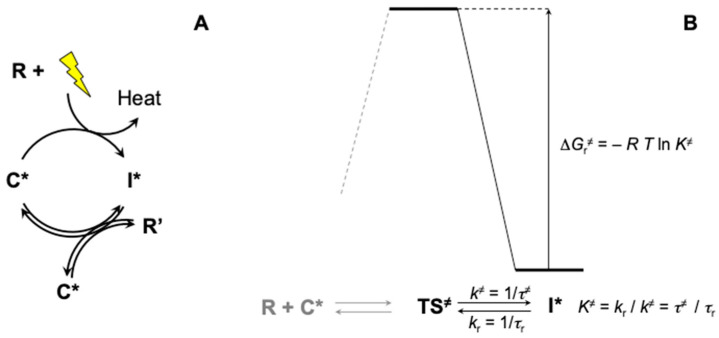
The potential that needs to be dissipated in order that the system becomes irreversible is assessed by considering a hypothetical reproduction cycle corresponding to the definition of an EAS in which the irreversible process (dissipation) is separated from the equilibrium synthesis of two copies of the catalyst C*. (**A**) Dissipation takes place during the formation of an intermediate I* from a catalyst C*. The intermediate I* carries the chemical potential needed for the formation of two copies of the unstable catalytic entity C*. **R** and **R’** constitute non-activated reactants. (**B**) Irreversibility can be accounted for by uniquely considering the transformation from the intermediate I* back to the transition state **TS^≠^**, which must be slower than the rate at which the whole cycle proceeds in the forward direction. It is independent of the values of free energy (omitted) characterizing states preceding the transition state **TS^≠^**, as the reactant state (**R** + C*, depicted in grey), which have been intentionally ignored to make this point unambiguous. The kinetic barrier Δ*G*_r_^≠^ is related to the ratio of the forward and reverse reaction rates (*K*^≠^ = *k*_r_/*k*^≠^) and therefore to that of the times needed for these processes to take place, namely *τ*^≠^ the lifetime of the transition state (defined as *τ*^≠^ = 1/*k*^≠^) and *τ*_r_ the timescale of the backward process (defined as *τ*_r_ = 1/*k*_r_).

The fact that biological development needs to be associated with dissipation, the result of irreversible processes, has the important consequence that the cell cycle itself develops in an irreversible way (Figure 2). This analysis is consistent with the irreversible character of biological reproduction [4,22]. The conditions for establishing an irreversible process can be assessed by considering an idealized reproduction cycle (Figure 5). This approach allows a conceptual separation of the system’s potential energy into two components, one which is dissipated as heat, and one which is needed to produce chemical work in synthesizing two copies of the catalyst C*. Kinetic irreversibility can then be achieved provided that the period of time *τ*_r_ (defined as 1/*k*_r_) required for reaching the transition state **TS^≠^** back from the intermediate I* exceeds the turnover time *τ*_cycle_ associated with the overall autocatalytic cycle (the reciprocal of turnover frequency, TOF [57], which is equivalent to the generation time for reproduction). Transition State Theory was formulated considering the two hypotheses, (*i*) that transition states can be considered at equilibrium with reactants, and (*ii*) that their breakdown is associated with a bond vibration along the mode leading to products so that the transition state lifetime is related to the period of the vibration (*τ*^≠^ ≈ *h*/*k*_B_ *T* corresponding to a value of ca. 1.6 × 10^−13^ s at 25 °C). A lower boundary for the free energy barrier Δ*G*_r_^≠^ can therefore be calculated by considering the inequality *τ*_r_ > *τ*_cycle_ established above:

Δ*G*_r_^≠^ > −*RT* ln(*τ*^≠^/*τ*_cycle_) (2)

Numerically this treatment leads to kinetic barriers Δ*G*_r_^≠^ exceeding values of 73 and 127 kJ mol^−1^ at 25 °C considering generation times spanning from 1 s to 1 century, respectively. A mean value of ca. 100 kJ mol^−1^ is obtained, corresponding to a 1-day generation time. These values are in agreement with earlier estimates based on a less straightforward treatment [83,84]. 

Irreversibility implies a negligible occurrence of the process for reaching **TS^≠^** back from I* meaning that thermal energy is not sufficient for promoting this conversion on the timescale at which the autocatalytic cycle proceeds. Owing to the principle of microscopic reversibility, the involvement of a slow thermal process of activation in the reverse direction means that the entire potential of **TS^≠^** should be dissipated as heat in the forward direction from **TS^≠^** to I*. This remark essentially illustrates how dissipation becomes a consequence of kinetic irreversibility. As expressed in Equation (2), the constraint on the kinetic barrier Δ*G*_r_^≠^ is rather related to time (the ratio of the duration of two events, namely transition state lifetime and reproduction timescale) than to thermodynamics. This barrier is required to ensure that irreversibility is achieved both on the scale of the reproduction of the macroscopic entity and at the microscopic scale (transition state of the rate-determining step of replication). Thermodynamic state functions associated with reactants are not involved in this calculation, which emphasizes the fact that kinetics is of paramount importance in self-organization. This observation is consistent with the early views of Lotka regarding the physics of life [85], when he stated that the laws of thermodynamics ‘*tell us that certain things cannot happen, but they do not tell us what does happen’*. The statement suggests that within the wide domain that is not forbidden by thermodynamics, extra-thermodynamic factors may turn out to be crucial in governing the actual evolution of the system.

## 4. Generality of the Model

Cellular processes are clearly more complex than the simple cycle of reproduction of the chemical autocatalyst in Figure 4D. Any chemical system made of low-molecular-weight derivatives will only allow a few possibilities for variation and evolution, considering the structure of the catalyst C*. However, and in spite of this limitation, evolutionary possibilities for these kinds of systems require additionally that C* be unstable. Such instability is necessary, otherwise, C* would not be driven to extinction through natural selection following the appearance of improved variants, as the catalyst would then remain in equilibrium with inert products. Furthermore, its instability would be of a thermodynamic kind and would come either from enthalpic (of high energy content) or entropic (of low probability) contributions. But once this condition is fulfilled, the entire cyclic system reaches a DKS state rather than just some of its components, thereby manifesting rudimentary autonomy within an EAS. The metabolic role of dissipation is assigned to avoid any violation of the Second Law, but it is hardly distinguishable from the synthetic part leading to reproduction (Figure 2), which necessarily becomes irreversible. That understanding leads to the more general understanding that the origin of life also requires the cooperation of different sub-systems [42].

Although the present report is limited by the intrinsic simplicity of the selected model, it reaches the original conclusion that irreversibility in the autocatalytic loop is related to the dissipation of an energy potential defined by the ratio of the timescales at which the two processes of reproduction and transition state breakdown proceed, as quantitatively expressed in Equation (2). Whether this analytical approach can withstand an extension towards more complex systems remains open to further investigation. More complex systems are likely to be associated with lower probabilities, corresponding to an additional decrease in entropy. This decrease could be compensated for by the dissipation of an increased amount of energy. Whether this compensation requires further dissipation or can come from thermodynamic stabilization involving, for example, molecular recognition remains unclear at this stage. However, modern biochemistry provides examples of highly complex strategies to circumvent this issue that emerged during the evolutionary process, for example, via the manner in which ATP is produced in the cell by generating and exploiting proton gradients across the cell membrane [86]. Such systems are so complex that the need for dissipation and its relationship with reproduction are concealed in present-day organisms, even though there is no argument that life is necessarily associated with the production of heat.

Thus, DKS is inherently connected with dissipation since it characterizes states that are held in an out-of-equilibrium state through fueling, whether by chemical or physical sources of energy. However, there is no direct evidence that a simple DKS system could develop strategies to increase this ability by better harvesting energy from available sources of energy. Replicating DKS systems has a particular advantage in this regard since evolution will conserve modifications that increase the persistence of the system while concurrently leading others to extinction [16,18]. Replicating systems able to undergo heritable modifications must therefore evolve in the direction of increased DKS in an environment able to provide matter and energy. It should therefore not be considered that an increase in dissipation is the driving force for evolution, but rather that it is due to an increase in DKS. Any increase in dissipation would be better understood as resulting from an improved kinetic scheme rather than from dissipation as a driving force in itself, a conclusion already reached by Astumian and co-workers [40]. This statement is confirmed by the fact that the introduction of a parasite in an autocatalytic cycle provides an example in which an increase in time stability was associated with a *decrease* in dissipation [39]. As in evolutionary biology, the emergence of new functions should not be viewed as the direct result of a driving force but rather as sorting among possible changes for those that are *kinetically* beneficial for the system. This contribution of contingency rather than direct determinism constitutes one of the major obstacles in the field, which has been expressed recently [24] as: “*Probably the greatest challenge is to manage state space and experimental conditions such that evolution becomes open-ended and the system repeatedly invents new functions. The search for open-ended evolution in a synthetic system is one of the few problems for which theory is unlikely to provide much guidance. Indeed, how does one allow a simulation to make inventions?*”.

More generally, the connection established here between metabolism and evolutionary features supports the claim that so-called metabolism-first or gene-first approaches should no longer be considered tenable in the origin of life field [87,88,89]. Establishing this linkage between metabolism and evolutionary features requires metabolism not to be reduced to its anabolic portion [26]. Indeed, Lotka proposed introducing natural selection as a physical principle [85] to account for life in physical and chemical terms. The relationships developed in this work demonstrate that such a goal also requires us to consider how energizing living organisms can induce their ability to undergo an evolutionary process.

Accordingly, this analysis focuses on the paramount importance of kinetics, consistent with the recent conclusions of Mandal et al. [40] that a dissipative system evolves to increase its kinetic stability. The fact that suppressing a process that is detrimental to evolution needs dissipation to be established and that this control is related to a potential determined by the ratio of two timescales emphasizes the importance of matching the timescales of the processes that contribute to the development of a dissipative system able to reproduce itself. This observation probably includes the rates of the different chemical steps as well as the timescales of the processes that take place in the environment [90]. The fact that the timescales of enzymatic biological processes span a much more limited range compared to their spontaneous non-catalytic counterparts [91] also suggests that matching timescales strongly facilitate biological processes.

An unexpected result of this analysis is that the DKS-stable system of Figure 4D presents a specific behavior associated with the need for an initiator related to energy feeding that may or may not be of a different nature from the disequilibrium responsible for the maintenance of the system. This observation means that both the situation in which the system is present and the one in which it is not may be kinetically stable. This fact, which actually corresponds to a situation analogous to that of living organisms with respect to the environment, may not be fortuitous and may have important consequences for the emergence of life, thereby orienting investigations towards the study of unstable rather than stable molecular components. The model in Figure 4D additionally indicates that a kinetically stable system can be composed of just energized species and become autonomous by avoiding the need for continuous initiation. This EAS, associating components of a system that undergo both destruction and regeneration and the maintenance of a non-equilibrium situation, is reminiscent of concepts put forward to account for life-like homeostasis and autopoiesis to characterize autonomous systems [92,93,94]. A consequence of these properties is that the whole EAS defines an inside/outside separation defined by the dependent state of the whole system with respect to its environment. Changes in the system can act on this dependence state, which, coupled to evolutionary processes, can be analyzed as seeds of cognition, opening a perspective which suggests that the gap between the physical sciences and the non-material facets of life can begin to close [95,96,97]. Moreover, the peculiarity of EASs like the one in Figure 4D, corresponding to kinetically stable situations, whether the system is present or not, was noted above. An unexpected consequence of this peculiarity is that the piece of information associated with the presence of the EAS in a non-equilibrium environment is reproduced along generations of intermediates of the cycle C*, I_1_* or I_2_* (Figure 4D). This statement suggests that information that is fundamentally epigenetic in nature and ontologically associated with all living organisms can exist. Thus, such information, transmittable across generations, may well originate in the kinetic properties of thermodynamically unstable but kinetically stable replicative chemical systems, rather than just within traditional genetic (genomic) sequences. Thus, in addition to the conventional representation of life as a process of reproduction with modification, this work illustrates the difficulty in accounting more deeply for this process as it relies on entangled and somewhat contradictory kinetic constraints of different origins that may contribute to the occurrence and stability of the replicative chemical system. Accordingly, the following elements form the basis of the analysis:

(i) Stoichiometrically originating constraints: more than one unit of the entity must be produced through the reproduction cycle, meaning that detrimental processes must proceed more slowly than reproduction.
(ii) Thermodynamically originating constraints: self-organization needs to be entropically compensated for through dissipation, which implies a non-equilibrium environment and kinetic irreversibility.
(iii) Evolutionary constraints: less efficient self-reproducing entities are driven to extinction, and their probability of being spontaneously reconstituted by chance must be limited by the timescale at which evolution proceeds.
(iv) Systems originating constraints: the system must function as a whole, leading to the emergence of system autonomy, meaning that the timescales of the different chemical processes need to be functionally compatible.

## 5. Conclusions

This work has been aimed at addressing the role of dissipation in inducing irreversibility in simple models of autocatalytic cycles without initially addressing the issue of evolvability. Maintaining an out-of-equilibrium state turns out to be a central facet of metabolism beyond the established ones of producing life’s building blocks and the high-energy currencies for driving thermodynamically unfavorable processes. The present analysis emphasizes that one of metabolism’s more important roles is to ensure a continuing connection between reproduction and dissipation. It is as a result of that connection that natural selection can become an efficient process for uncovering new functions in an open-ended evolutionary process [23]. By maintaining a disequilibrium state through the harvesting of available energy sources from the environment, metabolism ensures such evolutionary possibilities. A non-equilibrium environment by itself is not sufficient to promote self-organization, as shown by the intractable mixtures resulting from thermodynamically directed abiotic organic processes [7,8,9,10]. However, the onset of an EAS in such an environment would likely divert part of the flux of matter toward self-organization. Indeed, such an event would constitute an important breakthrough in the emergence of life process in that it would open the door to non-limited growth of a system in a DKS state, while the linkage with dissipation would allow the possibility of a further evolutionary process. Chemically, this event might ideally correspond to the contingent formation of *a single molecule* of one of the activated components of the EAS. Regardless, at this stage, we chose to focus on general properties by analyzing the behavior of minimal models. It is, however, worth mentioning that such analyses associated with the evolutionary contribution of metabolism in no way preclude the participation of amphiphiles or of precursors or derivatives of information carriers. To the contrary, such participation would be able to account for the simultaneous emergence of some of the systemic features of life [98].

In sum, the question of the origin of life does not primarily turn out to be based on synthesizing particular biomolecules but rather on establishing systems in which kinetic factors become paramount [39]. It does not invalidate a reductionist approach to understanding life but, on the contrary, supports one that includes a contribution pertaining to the kinetic behavior of energized thermodynamically unstable intermediates, as opposed to one involving stable prebiotic building blocks. The kinetic nature of the coupling of dissipation with selection as a process that enhances DKS ensures that self-organization can proceed irreversibly, becoming possible through the relationship between energy, transition state lifetime, and generation time, with the important consequence that autonomous chemical systems may emerge naturally. Though the analysis of systems more complex than the rudimentary EASs described here may lead to refinement of these broad conclusions, it is unlikely to totally dismiss the role of these key parameters. Furthermore, this work additionally confirms that uncovering how life’s first emergent steps took place will likely also make life’s physicochemical principles easier to understand [16]. It suggests that progress can be made in this direction by considering models that are relatively simple. Through such approaches, the constraints governing the survival and evolvability of the first living organisms, hidden deep within the complex processes that evolution progressively built up to circumvent those constraints, may be revealed.

## Data Availability

All study data are included in the article.
